# Genetic Diversity and Population Genetic Structure Analysis of *Echinococcus granulosus* *sensu stricto* Complex Based on Mitochondrial DNA Signature

**DOI:** 10.1371/journal.pone.0082904

**Published:** 2013-12-09

**Authors:** Monika Sharma, Bashir Ahmad Fomda, Saligram Mazta, Rakesh Sehgal, Balbir Bagicha Singh, Nancy Malla

**Affiliations:** 1 Department of Parasitology, Post Graduate Institute of Medical Education and Research, Chandigarh, India; 2 Department of Microbiology, Sher-i Kashmir Institute of Medical Sciences, Srinagar, Kashmir, Jammu and Kashmir, India; 3 Department of Community Medicine, Indira Gandhi Medical College, Shimla, Himachal Pradesh, India; 4 School of Public Health and Zoonoses, Guru Angad Dev Veterinary and Animal Sciences University, Ludhiana, Punjab, India; Nanjing Medical University, China

## Abstract

The genetic diversity and population genetics of the *Echinococcus granulosus* *sensu stricto* complex were investigated based on sequencing of mitochondrial DNA (mtDNA). Total 81 isolates of hydatid cyst collected from ungulate animals from different geographical areas of North India were identified by sequencing of cytochrome *c* oxidase subunit1 (*coxi*) gene. Three genotypes belonging to *E. granulosus* *sensu stricto* complex were identified (G1, G2 and G3 genotypes). Further the nucleotide sequences (retrieved from GenBank) for the *coxi* gene from seven populations of *E. granulosus* *sensu stricto* complex covering 6 continents, were compared with sequences of isolates analysed in this study. Molecular diversity indices represent overall high mitochondrial DNA diversity for these populations, but low nucleotide diversity between haplotypes. The neutrality tests were used to analyze signatures of historical demographic events. The Tajima’s D test and Fu’s FS test showed negative value, indicating deviations from neutrality and both suggested recent population expansion for the populations. Pairwise fixation index was significant for pairwise comparison of different populations (except between South America and East Asia, Middle East and Europe, South America and Europe, Africa and Australia), indicating genetic differentiation among populations. Based on the findings of the present study and those from earlier studies, we hypothesize that demographic expansion occurred in *E. granulosus* after the introduction of founder haplotype particular by anthropogenic movements.

## Introduction

Cystic echinococcosis, an infection with the metacestode of the dog tapeworm *Echinococcous granulosus*, is a global public health problem that infects the human and ungulate animals [1]. The life cycle of *E. granulosus* involves dogs and other carnivores as definitive host and livestock as intermediate host [2]. Eggs shed in the feces of definitive host are ingested by intermediate host where they develop into metacestode stage and establish hydatid cysts. Although the Infection in livestock is usually asymptomatic and detected during post mortem examination at the slaughter houses, yet it causes economic loss through condemnation of infected organ [3]. 

*E. granulosus* is a complex of distinct strains with different host affinities. To date, 10 different genotypes have been described by molecular genetic analysis and the genotypic variation closely follows the biological and phenotypic characteristic of the parasite. These 10 genotypes/strains are named on the basis of their ideal intermediate host. These include sheep strain (G1), Tasmanian sheep strain (G2), buffalo strain (G3), horse strain (G4), cattle strain (G5), camel strain (G6), pig strain (G7), cervid strain (G8), pig/human strain (G9) and Fenno-Scandian cervid strain (G10) [4–6]. These genotypes can infect different ungulates in spite of their ideal intermediate host. It has been proposed that *E. granulosus* genotype should be split into different species: *E. granulosus* *sensu stricto* (genotypes G1-G3), *E. equinus* (genotype G4), *E. ortleppi* (genotype G5), *E. canadensis* (genotype G6-G10) and *E. felidis* (lion strain) [7–9]. Strain variation of *E. granulosus* reflects the differences in life cycle pattern and host range; thus the knowledge of genetic diversity and population genetics of this parasite is of immense public health importance for formulation of control strategies for hydatidosis. In Western India, 4 different genotypes (G1, G2, G3 and G5) were reported in different intermediate hosts such as cattle, buffaloes, pigs and sheep [10]. Barring the reports published by Singh et al. [11] from Ludhiana (North India) on 10 isolates of *E. granulosus* and found the G1 and G3 genotype by *cox1* gene sequencing, an extensive study on the genotypes of parasite involving large number of isolates and covering large geographical endemic areas is lacking in this region.

 Mitochondrial DNA has shown to be useful in differentiation of all the genotypes of *E. granulosus* and acts as an important genetic marker to study the population genetic structure of this parasite as it is haploid, non recombining, multicopy, rapidly evolving and maternally inherited. The rate of evolution of mitochondrial genome appears to be exceeding from that of single-copy fraction of nuclear genome. The high rate may be due to an elevated rate of mutation in mitochondrial DNA, because of this high rate of evolution, mitochondrial DNA is likely to be useful for high-resolution analysis of the evolutionary process [12]. The aim of present study was to genotype the North Indian animal isolates of *E. granulosus* by sequencing of mitochondrial *cox1* gene. The results were further compared with nucleotide sequences retrieved from GenBank from other geographical regions to study the genetic variability and population genetics of this parasite.

## Materials and Methods

### Ethics statement

The study was approved by the Institutional Ethics Review Committees of the Post Graduate Institute of Medical Education and Research (PGIMER), Chandigarh, Sher-i- Kashmir Institute of Medical sciences (SKIMS), Srinagar, Kashmir and Indira Gandhi Medical College and Hospital (IGMC), Shimla, India. In this study hydatid cysts were collected from animals during post-mortem examination following inspection by the veterinary officers at the respective slaughter houses with their due consent. No experimentation was done on animals, therefore no approval from Institutional animal ethics committee was required.

### Sample collection and Molecular analysis

Hydatid cysts were collected randomly during 2010-2012 from 4 different geographical areas in North India. The numbers depend upon the availability of the sample. Cyst samples were obtained from 74 freshly slaughtered and heavily infected sheep from slaughter houses, falls under the jurisdictions of the municipal corporation of Chandigarh (n=66), Shimla, Himachal Pradesh (n= 3) and Srinagar, Jammu and Kashmir (n=5). Permission was obtained from these slaughter houses to use these hydatid cysts. Seven hydatid cysts were kindly received from Guru Angad Dev Veterinary and Animal Sciences University, Ludhiana, Punjab, North India. These cysts were isolated from sheep (n=2), buffaloes (n=2), goats (n=2) and cow (n=1). Thus, in total isolates from 81 animals were analyzed. The cysts were transported to the Department of Parasitology, Post Graduate Institute of Medical Education and Research (PGIMER), under refrigerated conditions for further processing. Intact hydatid cysts were separated and washed with distilled water. Cyst from each animal was considered as an isolate.

 Cyst samples were washed thrice in PBS to remove ethanol and genomic DNA was extracted from each sample by QIAamp DNA mini kit (Qiagen, Hilden, Germany), according to the manufacturer’s instructions. For molecular identification, PCR amplification of the *cox1* gene was performed by using primers and PCR conditions as described previously [4] with minor changes [13]. To avoid the amplification of pseudogene, BLAST search was performed with the primer sequence used. Positive, negative and internal controls were also used. Briefly, amplification was performed in 50 μl final volume containing 2 μl DNA, 0.2 mM premixed solution of dNTP, 10 pmol of each primer, 1x PCR buffer and 1 U of TaqDNA polymerase. Amplification program included an initial denaturation step of 95°C for 5 min and 38 cycles each of denaturation (95°C for 50s), annealing (57°C for 50s), extension (72°C for 1 min) and final extension of 72°C for 10 min. After agarose gel electrophoresis (1.5%), PCR products were purified and sequenced.

### Comparative analysis of the nucleotide sequences of isolates used in the present study with sequences from other countries deposited in GenBank

#### Phylogenetic analysis

Different sequence of *E. granulosus* *sensu stricto* population deposited in the GenBank from India and other South Asian, East Asian, European, Middle East, African, South American and Australian countries were retrieved from the National Center for Biotechnology (http://www.ncbi.nlm.nih.gov) and compared with sequences of isolates used in the present study (Table 1). For this sequences for the *cox1* gene were retrieved and all these sequences should contain the differential nucleotide positions on the basis of which 10 genotypes of *E. granulosus* have been defined. Nucleotide sequence analysis was performed with BLAST sequence algorithms and sequences were aligned using ClustalW [14]. Samples were clustered using the PhyML [15] as part of SeaView v. 4.2.4 [16].

**Table 1:** Accession numbers of nucleotide sequenced of *cox1* gene of *Echinococcus granulosus* *sensu stricto* from different countries.

**S. No. **	**Country**	**N**	**Accession number**
1	**South Asia**	**103**	
	North India	88	JX854022 (n=2), JX854024 (n=3), JX854025 (n=1), JX854028(n=53), JX854029(n=13) JX854030 (n=1), JX854032(n=1), JX854033(2), JX854034 (n=8), KC422644(n=1), KC422645(n=1), KC894722(n=1), KC894723(n=1)
	East India	12	DQ269942-47, DQ333184-86, DQ104330-31, DQ109036
	Nepal	3	AB522646-47, AB551110
2	**East Asia**	**46**	
	China	46	AB491441, AB491443- 44, AB491446, AB491450, AB491453, AB491456, AB688602-19, DQ356874-83, AY386206-07, JQ317991-98, JQ318001
3	**South America**	**51**	
	Argentina	13	FN564569, JN176927-31, GU980906-12
	Chile	29	GQ502213- 23, GQ502225-38, GQ502240-43
	Peru	9	JF828330-34, JF828336-37, AB688620-21
4	**Europe**	**51**	
	South of France	5	JQ356711- 15
	Austria	12	AJ508016-19, AJ508021-28
	Portugal	24	FN646353-58, FN646361-75, FR666904-05, AJ508020
	Italy	2	FJ608752, FJ608726
	Hungary	1	JF690976
	Greece	2	DQ856466-67
	Romania	5	AY686561, AY686563, AY686559, JF520817-18
5	**Middle East**	**94**	
	Iran	39	FJ796205, FJ796206, HM563001-08, HM563010-11, HM563015-17, HM563022, HM130577, HM130582-83, HM130597-98, HM626405, JN604104-5, JX087363, JQ250806-17, JQ219963-64,
	Iraq	5	JX878689- 93
	Jorden	12	AB688590- AB688601
	Palestine	20	KC109641-60
	Turkey	18	AJ508011-15, HM598451-52, JQ031131, JN810792, GU951512-13, EU178105, EF545563, HQ717148-50,HQ717156, HQ703429
6	**Africa**	**19**	
	Tunisia	1	AY850565
	Morocco	9	EF367293-94, EF367263-65,EF367254, EF367242-43, EF367291
	Ethiopia	6	AB650529- 34
	Libya	3	HM636639- 41
7	**Australia**	**5**	AJ508029-32, AJ508010

N=Total number of sequences

#### Gene genealogies

Identification of haplotypes and their networks was constructed, based on parsimony criteria [17] using the TCS version 1.2 software [18]. The network estimation was run at 95% probability limit. This haplotype network analysis is useful for intraspecific data in revealing multiple connections between haplotypes and indicating possible missing mutational connections. 

#### Population genetic analysis

For population genetic analysis, the nucleotide sequences of all the isolates used in the present study together with sequences retrieved from GenBank were grouped in 7 populations: South Asian, East Asian, Middle East, European, African, South American and Australian. Population diversity indices such as numbers of segregating sites (S), haplotypes number (h), haplotype diversity (Hd) and nucleotide diversity (π) and average number of pairwise nucleotide differences within population (K), were estimated using DnaSP 4.5 Software [19]. The neutrality indices of Tajima's D [20] and Fu's Fs [21] in each population were also calculated. 

 The pairwise genetic difference was estimated for all populations by calculating Wright’s F-statistics (Fst) and gene flow (Nm) was calculated by population genetics package Arlequin 3.1 [22]. In addition, average number of pairwise nucleotide differences (Kxy), nucleotide substitution per site (Dxy), and net nucleotide substitution per site (Da) between populations were also calculated by DnaSP. 

## Results

**Figure 1:**
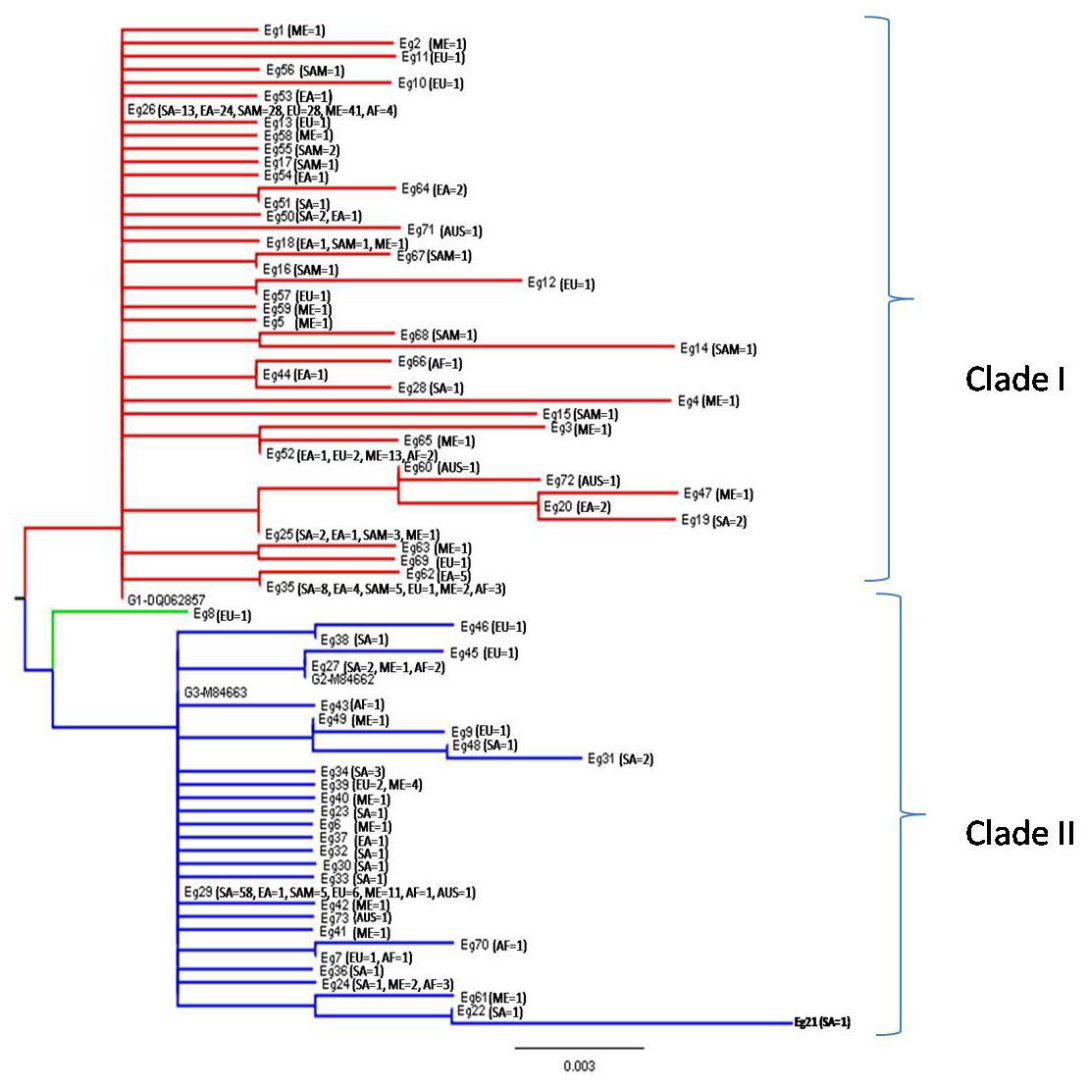
Dendrogram constructed from the coxI sequences of *E. granulosus* isolates analysed in the present study along with those retrieved from GenBank. The 369 gene sequences are clustered in 73 haplotypes (Eg1-Eg73). SA: South America; EA: East America; EU: Europe; ME: Middle East; AF: Africa; SAM: South America; AUS: Australia.

The amplification of *cox1* gene with JB3/JB4.5 primer yielded PCR product of 446 bp. Nucleotide sequences of all the 81 isolates from North India analyzed in the present study were aligned with reference sequences of each genotype within *E. granulosus* retrieved from GenBank. Total 3 genotypes of *E. granulosus* were found: G3 genotype (n=58), G1 genotype (n=22) and G2 genotype (n=1). The various sequences of the haplotypes found in this study, were deposited in GenBank with accession numbers JX854022-34 and KC422644-45. Sequences of these isolates along with those retrieved from GenBank were used to construct a phylogenic tree (Figure 1). Among 369 sequences, total 73 sequence variants (named as Eg1 -Eg73) were grouped in two main clades. Clade I comprises G1 genotype and its microvariants, and Clade II comprises G3 genotype and its microvariants. The G2 genotype is also clustered in Clade II. 

### Gene/allele genealogy

**Figure 2:**
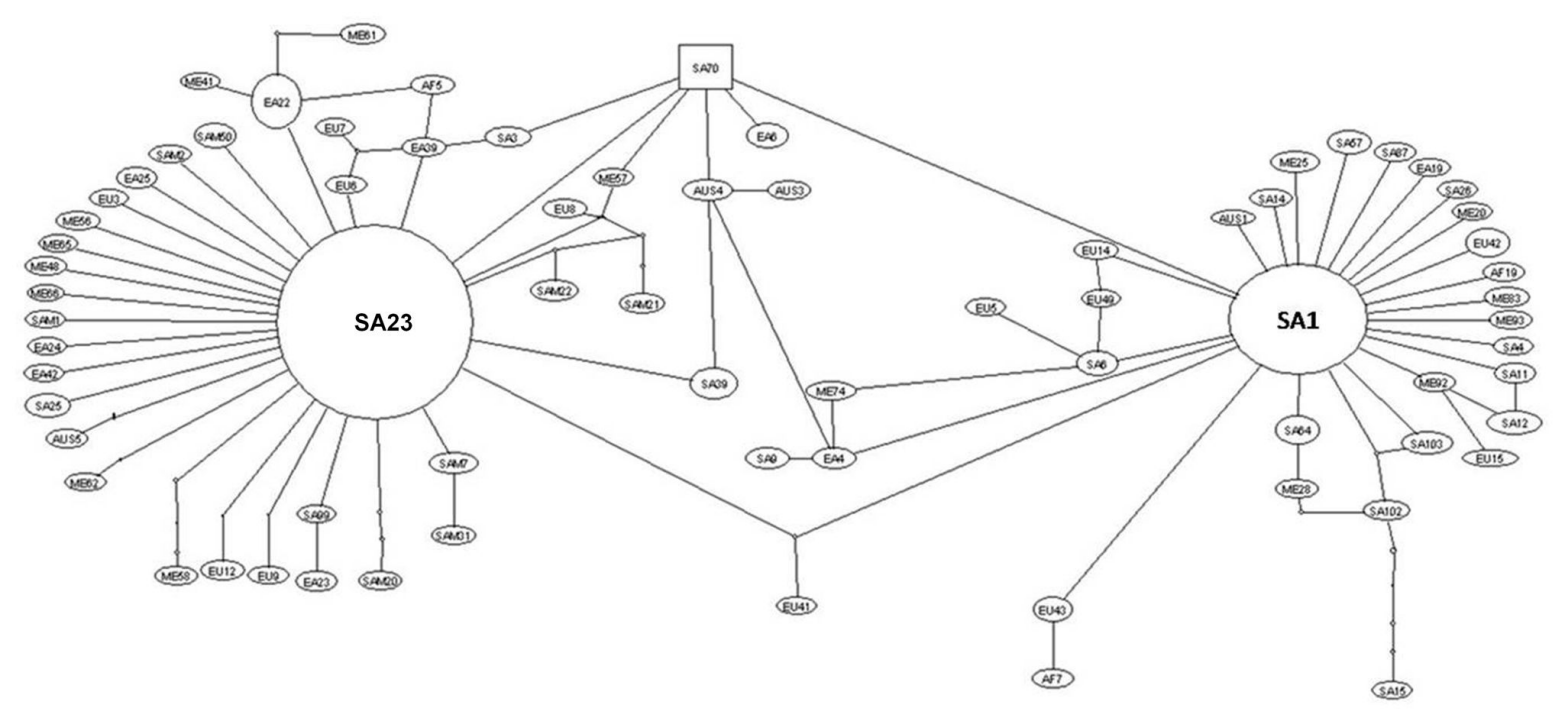
Haplotype network of *cox1* gene of *Echinococcus granulosus* *sensu stricto* complex. Major circles represent predominant haplotypes. A branch represents a single nucleotide change and dots on branches represent inferred missing haplotypes. SA: South America; EA: East America; EU: Europe; ME: Middle East; AF: Africa; SAM: South America; AUS: Australia.

The genealogic relationships among the *cox1* sequences estimated by TCS software detected two common haplotypes (SA1 and SA23) (Figure 2). The SA23 haplotype was reported in European (54.9%), South American (54.9%), East Asia (52.1%), Middle East (45.7%), African (42.1%) and South Asian (12.6%) populations and SA1 haplotype reported in South Asian (56.3%), Australian (20%), Middle East (11.7%), European (11.7%), South American (9.8%), African (5.2%) and East Asian (2.1%) populations. Thus the both haplotypes shared wide geographical distribution; SA23 was predominant in Middle East, European, South American populations, whereas the SA1 was predominant in South Asian population. These two common haplotypes showed many single haplotypes around it and star-like shape of this part of network indicates a high frequency of the unique mutations, which can be an indication of rapid population expansion.

### Nucleotide polymorphism

 Among 369 sequences (retrieved from GenBank and sequences of isolates analyzed in this study), total 73 haplotypes were found: 20 in South Asia, 14 in East Asia, 17 in Europe, 25 in Middle East, 10 in Africa, 13 in South America, 5 in Australia (Table 2). Along the 341 bp reference alignment, only nucleotide substitutions were detected and insertions or deletions were not detected. Total 67 point mutations were noted and 17 were parsimony informative sites.

**Table 2:** Diversity and neutrality indices of *Echinococcus granulosus* *sensu stricto* population calculated from nucleotide sequence of mitochondrial *cox1* gene.

**Geographical origin**	**n**	**S**	**K**	**H**	**Hd ± S.D**	**π ± S.D**	**D**	**Fu’s Fs**
SA	103	22	1.334	20	0.664±0.050	0.0039 ±0.0005	-2.100*	-15.396**
EA	46	13	1.373	14	0.716±0.070	0.0040±0.0006	-1.629*	-9.286**
EU	51	20	1.739	17	0.690±0.071	0.0051±0.0007	-1.935*	-11.269**
ME	94	23	1.748	25	0.761±0.040	0.0051±0.0004	-1.816*	-20.684**
AF	19	8	2.22	10	0.918±0.036	0.0065±0.0007	-0.099	-4.311**
SAM	51	17	1.27	13	0.685±0.069	0.0037±0.0006	-2.060*	-7.917**
AUS	5	7	3.20	5	1.000±0.126	0.0093±0.0021	-0.331	-2.116
TOTAL	369	67	1.82	73	0.799±0.017	0.0053±0.0002	-2.462*	-111.06*

Statistical significance: * P < 0.05; ** P < 0.01

SA: South Asia; EA: East America; EU: Europe; ME: Middle East; AF: Africa; SAM: South America; AUS: Australia.

n: number of sequences examined; S: Number of segregating (polymorphic/variable) sites; K: Average number of pairwise nucleotide differences; H: Number of haplotypes; Hd: Haplotype diversity; π: nucleotide diversity; D: Tajima's D test statistics

### Diversity indices

Population genetic indices were calculated using the nucleotide data of *cox1* gene from India and other countries (Table 2). Haplotype diversity (Hd) for all the 369 sequences was calculated to be 0.799 +/- 0.017 SD. Average number of nucleotide differences, k was found to be 1.82 and nucleotide diversity (π) was 0.0053 +/- 0.0002. Haplotype and nucleotide diversity indices were highest in Australian population followed by African population and lowest in South Asian population. Neutrality Indices calculated by Tajima’s D and Fu’sFs test were negative in all populations, the D value was significantly negative in all except African and Australian populations. Fu’sFs value was significantly negative in all except Australian population.

Inter-population nucleotide differences (Kxy) and average number of nucleotide substitutions per site between all these populations (Dxy) varied from 1.33 and 0.00391 (East Asia and South America) to 2.8 and 0.00821(Africa and Australia) respectively (Table 3). Pairwise genetic distance (Fst) in all the seven populations varies from - 0.00201 with Nm value=infinite (between Europe and Middle East) to 0.37828, Nm= 0.82176 (between South Asia and South America) (Table 4). The Fst value between Europe and Middle East and Gst value between Europe and South America were found to be negative, indicating no differentiation at these loci [23]. When population of South Asia was compared with other populations the value of Fst range from 0.10273 to 0.37828 with Nm value range 0.82176 to 4.36727 indicating these populations are differentiated with low gene flow. Middle East countries in comparison to other countries show very low genetic differentiation (Gst 0.00184 to 0.10203, Fst -0.00201 to 0.25395) with very high gene flow (Nm 1.46891 to infinite). Further, except between Europe and Middle East, South America and East Asia, South America and Europe, Africa and Australia, all other populations showed significant pairwise genetic distance. 

**Table 3:** Population genetics indices between different populations of *Echinococcus granulosus* *sensu stricto* calculated from nucleotide sequence of mitochondrial *cox1* gene.

**Population1**	**Population2**	**Kxy^*a*^**	**Dxy^*b*^**	**Da^*c*^**	**Gst^*d*^**
South Asia	East Asia	2.07	0.00608	0.00211	0.12444
South Asia	Europe	2.05	0.00601	0.00151	0.10984
South Asia	Middle East	2.06	0.00605	0.00153	0.10203
South Asia	Africa	1.92	0.00565	0.00044	0.05540
South Asia	South America	2.10	0.00618	0.00235	0.11352
South Asia	Australia	2.35	0.00690	0.00025	0.04798
East Asia	Europe	1.59	0.00467	0.00011	0.00383
East Asia	Middle East	1.61	0.00475	0.00017	0.00968
East Asia	Africa	2.11	0.00619	0.00092	0.03202
East Asia	South America	1.33	0.00391	0.00002	0.00065
East Asia	Australia	2.60	0.00765	0.00094	0.06416
Europe	Middle East	1.74	0.00510	-0.00001	0.00184
Europe	Africa	2.15	0.00632	0.00051	0.03696
Europe	South America	1.54	0.00453	0.00010	-0.00170
Europe	Australia	2.76	0.00810	0.00085	0.06262
Middle East	Africa	2.11	0.00620	0.00038	0.01920
Middle East	South America	1.57	0.00462	0.00019	0.00777
Middle East	Australia	2.75	0.00808	0.00082	0.05166
Africa	South America	2.120	0.00624	0.00111	0.03544
Africa	Australia	2.80	0.00821	0.00026	0.03121
South America	Australia	2.64	0.00776	0.00120	0.06414

a: Average proportion of nucleotide differences between populationsb: The average number of nucleotide substitutions per site between populations,c: The number of net nucleotide substitutions per site between populations,d: Genetic differentiation index based on the frequency of haplotypes.

**Table 4:** Pairwise genetic distance (Fst in lower diagonal) and gene flow (Nm in upper diagonal) between different populations of *Echinococcus granulosus* *sensu stricto* calculated from nucleotide sequence of mitochondrial *cox1* gene.

	**SA**	**EA**	**EU**	**ME**	**AF**	**SAM**	**AUS**
**SA**		0.93725	1.42252	1.46891	4.36727	0.82176	2.66392
**EA**	0.34789*		21.63745	14.49069	2.46858	91.89622	1.56101
**EU**	0.26008*	0.02259*		∞	5.13096	21.30169	2.17314
**ME**	0.25395*	0.03335*	-0.00201		6.56639	12.89370	2.15257
**AF**	0.10273*	0.16843*	0.08879*	0.07076*		1.93160	7.30769
**SAM**	0.37828*	0.00541	0.02293	0.03733*	0.20563*		1.21247
**AUS**	0.15803*	0.24260*	0.18705*	0.18850*	0.06404	0.29198*	

Statistical significance *P < 0.05.

SA: South Asia; EA: East America; EU: Europe; ME: Middle East; AF: Africa; SAM: South America; AUS: Australia.

## Discussion

Pednekar et al. [10] have reported four genotypes of *E. granulosus* namely G1 (sheep strain), G2 (Tasmanian sheep strain), G3 (Indian buffalo strain) and G5 genotypes (cattle strain) in livestock in Maharashtra and adjoining areas in Western India. The predominant genotype was found to be G3 (63%) present in all species of livestock followed by the G5 (19.56%), the G1 (13%) and the G2 genotype (4.34%). In earlier reports from Ludhiana (North India), only 2 genotypes, buffalo strain (G3) and common sheep strain (G1) were found to infect the livestock [11]. In our earlier study, genotyping of hydatid cysts from cystic echinococcosis patients from 7 different states of North India revealed the zoonotic potential of G1-G3 complex (94%), G5 (3.1%) and G6 (3.1%) genotypes of *E. granulosus* [13].

In the present study, 3 genotypes of *E. granulosus* were found to infect the livestock: G1, G2 and G3 genotypes. In concordant to the earlier studies from livestock (cattle, buffalo, pig and sheep) in India, the G3 genotype (71.8%) was found as predominant genotype. The second most common genotype was the G1 genotype in 27.16 % isolates followed by the G2 genotype in only one isolate from Srinagar, Kashmir (North India) which was similar to the findings in Eastern India [10]. Further, in contrast to the results of the present study, G1 was reported as dominant genotype in other countries: for example 95.74% in China [24], 87.5% in Iran [25], 77.4% in Southern Brazil (with 11.11% of G3 genotype) [26], 71.59% in Italy (with 27.8% prevalence of G3 genotype) [27], 66% in Turkey [28] and 55.8 % in Pakistan (with 44.11% prevalence of G3) [29]. This data suggest that while moving from Middle East to Europe, South America and South Asia, the prevalence of the G3 genotype started increasing and emerged as predominant genotype in South Asia while in East Asia the G1genotype emerged as predominant genotype. 

In the present study the G2 genotype was clustered in Clade II. The large numbers of *E. granulosus cox1* gene sequences for the G2 genotype, registered in GenBank or international database are infact the microvariants of G3 genotype. This discrepancy could be due to some *cox1* gene sequences for G2 genotype deposited in GenBank which were used as reference ones [27]. These reference ones partially differ in variable sites from those published by Bowles et al. [4] who had characterized the genotypes of *E. granulosus* on the basis of sequencing of *cox1* gene. 

To date, very few studies have explored in depth population genetic structure of *E. granulosus* [30–33]. These studies have shown *cox1* gene as a promising candidate for revealing the population genetics of *E granulosus*. In the present study, *E. granulosus* *sensu stricto* populations from wide geographical areas were analyzed to examine the parasite genetic diversity. For this, sequences of only *E. granulosus* *sensu stricto* complex (G1-G3 genotypes) were retrieved from GenBank/EMBL/DDBJ International Databases as there is scarcity of data in GenBank for other genotypes and also in the present study, *E. granulosus* *sensu stricto* complex was found as predominant species in livestock.

 In the present study, haplotype network has shown that two predominant haplotypes of *E. granulosus* *sensu stricto* (SA1 and SA23) are widely distributed in different geographical area. Interestingly, the nucleotide sequence of this haplotype (SA23) was 100% identical to previously described as predominant haplotype in Europe (EG1: JF513058) [32], China and Peru (G01: AB491414) [31], Iran and Jordan (EG01) [33]. The haplotype (SA1), found as second dominant genotype in Middle East, China, Europe and South America was predominant in South Asia. In dendrogram analysis, SAI haplotype was clustered with G3 genotype and SA23 with G1 genotype, indicating the G3 as predominant in Asian country and G1 in European, South American, African, European, East Asian and Middle East countries.

Despite the wide distributional range, the estimation of inter-population comparison (Kxy, Dxy, Gst and Fst) also support low to high level of genetic differentiation between these populations. The populations of Europe, Middle East and South America showed low divergence and shared the common haplotypes. The Europe and Middle East populations are highly closely related to each other which were suggested by a very low and non-significant Fst value. Gene flow (Nm) was also found to be very high between these two populations. The South Asian population was the most differentiated with very low gene flow among other populations. This could be related to the presence of G3 as predominant genotype in this population as demonstrated in dendrogram and haplotype network. 

In the present study, in spite of high haplotype diversity, low nucleotide diversity values suggested small differences between haplotypes. This was also demonstrated by the haplotype network, which represents mostly single nucleotide differences between majorities of haplotypes. In the present study, the combination of high haplotype and low nucleotide diversity can be a signature of a rapid population expansion from a small effective population size as observed in the earlier study [34]. A number of statistical tests have been developed to test selective neutrality of nucleotide variability and they are used to determine the population growth [35]. These tests are based on distribution of pairwise differences between nucleotide sequences within populations. In this study, Tajima’s D test and Fu’s Fs tests were applied that are usually used to find out the population expansion and differ slightly in their approach. The Tajima’s D test [20] is based on comparison of the allelic frequency of segregating nucleotide sites. A positive value of this test indicates a bias towards intermediate frequency alleles, negative value indicates a bias towards excess of the number of rare alleles and the latter being a signature of recent population expansion. Fu’s FS test [21] is based on the alleles or haplotypes distribution, and here too negative values can indicate an excess number of alleles, as would be expected from a recent population expansion or from genetic hitchhiking. In this study, Tajima’s D test was significantly negative for all except African and Australian populations. Fu’s FS test resulted in significant negative values for all except Australian population which was negative but not significant. The overall negative values of both neutrality tests indicate excess of the rare mutations in populations, which can imply the recent population expansion. Further analysis by including the additional neutral nuclear DNA markers could provide a more complete perspective on population genetic structure of this parasite.

The Interpretation of demographic expansion correlates well to the widely observed patterns of domestication of sheep which started around 12,500 B.C. The various genetic and archaeological evidences suggest that domestication of sheep occurred first in Southwest Asia (Middle East) and then spread successfully into Europe and Africa, and the rest of Asia [36]. The result of present study have suggested that parasite along with its intermediate host was introduced into Europe and Africa from Middle East and then to South America, Australia and other parts of Asia. Recently, a similar hypothesis regarding dispersal of parasite was proposed in European, South American and Middle East populations [32,33]. In the present study comparison of pairwise genetic distance showed that South Asian population is closely related to African population and the later is closely related to Middle East population which suggest the dispersal of parasite from Middle East to Africa and then to South Asia. The lack of the archaeoparasitological evidence does not allow us to infer how the parasite invaded South Asia. The population genetic structures of *E. granulosus* *sensu stricto* should be compared in different endemic areas to know about the ancestral origin and process of worldwide dispersal of this parasite.

In conclusion, molecular analysis demonstrates the presence of G3 as predominant genotype followed by the G1 and G2 genotype of *E. granulosus* in ungulate animals in North India. The present study reveals a high genetic diversity within populations of *E. granulosus* and but relatively low to high level of genetic differentiation among the populations. The observed patterns of genetic diversity within and between the populations are likely caused by population expansion after the introduction of founder haplotype. Finally, present study supports the earlier report that it is important to correlate molecular epidemiology with evolutionary biology so that population genetics and phylogenetic analyses are able to confer a considerable added value in the characterization of strains and species of pathogens [37].
